# Urine and Serum Electrolytes and Biochemical Values Associated with Osteoporosis in Premenopausal and Postmenopausal Women: A Longitudinal and Cross-Sectional Study Using Korean Genome and Epidemiology Study (KoGES) Cohort

**DOI:** 10.3390/jcm10102155

**Published:** 2021-05-17

**Authors:** Hae-Sang Park, Ga-Young Kim, Jong-Ah Lo, Jin-Sun Kim, Shin-Young Ahn, Gang-Jee Ko, Young-Joo Kwon, Ji-Eun Kim

**Affiliations:** 1 Department of Internal Medicine, Korea University Guro Hospital, Seoul 08308, Korea; judycrom@naver.com (H.-S.P.); kkay0803@gmail.com (G.-Y.K.); rohja3862@gmai.com (J.-A.L.); dalky35@naver.com (J.-S.K.); sypooh712@naver.com (S.-Y.A.); lovesba@hanmail.net (G.-J.K.); yjkwon@korea.ac.kr (Y.-J.K.); 2 Department of Internal Medicine, Korea University College of Medicine, Seoul 02841, Korea

**Keywords:** osteoporosis, menopause, electrolytes, longitudinal study, biomarker

## Abstract

Osteoporosis is a major public health concern, especially in women. This study aims to identify early biomarkers from biochemical measurements of serum and urine for recognizing the development of osteoporosis and osteopenia in premenopausal and postmenopausal women. From the Korean Genome and Epidemiology Study (KoGES) cohort, longitudinal study participants with normal bone density were enrolled and assessed for the association of baseline clinical and biochemical factors with osteoporosis development over 4 years. In addition, a cross-sectional study between normal bone density and osteopenia/osteoporosis was conducted to validate the risk factors found in the longitudinal cohort. Of the 5272 female participants in the KoGES cohort, 813 women (501 premenopausal and 312 menopausal) who had normal bone density at baseline were included in the longitudinal study. During the 4 years of follow-up, 64 patients developed osteoporosis and 354 developed osteopenia. In a multivariate logistic regression analysis, serum calcium and urine uric acid levels were significantly associated with elevated osteoporosis risk in premenopausal and postmenopausal women, respectively (risk of osteoporosis by serum calcium levels in premenopausal women: 4.03 (1.09–14.93), *p* = 0.037; risk of osteoporosis by urine uric acid levels in postmenopausal women: 24.08 (1.79–323.69), *p* = 0.016). For the cross-sectional study, serum and urine parameters were compared between women with osteopenia or osteoporosis at baseline and those with normal bone density. Urine uric acid levels were found to be significantly higher in both premenopausal and postmenopausal women with bone loss than in women with normal bone density (*p* < 0.001 and *p* = 0.004, respectively). Uric acid level in urine may be an early marker for the development of osteoporosis in women, especially after menopause.

## 1. Introduction

Osteoporosis is a major cause of broken bones in the elderly, and osteoporotic fractures can cause chronic pain and decrease performance in daily activities [1,2]. The World Health Organization (WHO) has estimated the lifetime risk for osteoporotic fractures of the hip, vertebra, or wrist to be 30–40% [3]. Osteoporosis and osteopenia were defined as decreased bone density compared to that in young adults, based on bone mineral density (BMD) assessment [3,4]. Osteoporosis is more common in women, especially after menopause, and changes in female sex hormones, such as estrogen, are thought to play an important role in bone health [5,6,7]. Since most of the studies to date have mainly focused on postmenopausal osteoporosis, though some studies have revealed the risk factors for variation in bone status in premenopausal women [8,9,10], the differences in risk factors for premenopausal and postmenopausal osteoporosis have not yet been clearly evaluated. In addition, despite the large number of studies attempting to identify various risk factors for bone loss, only a few have analyzed the correlation between urine and/or serum electrolytes, biochemical measurements, and the risk of osteoporosis [11,12,13]. The current study aims to identify the premenopausal and postmenopausal risk factors of bone loss in urine and serum electrolytes and biochemical values using longitudinal and cross-sectional cohorts.

## 2. Materials and Methods

### 2.1. Study Setting and Study Cohort

This was an observational analysis using a community-based prospective cohort recruited from the Korean Genome and Epidemiology Study (KoGES). The KoGES cohort consisted of 10,030 individuals aged 40–69 years, who were residents of either Ansan (urban area) or Anseong (rural area) in Gyeonggi-do, Korea. Continuous health screenings and surveys were conducted every two years since 2001. A description of how the KoGES cohort was established can be found in the published literature [14].

From the baseline survey in 2001 to the 7th follow-up survey of the KoGES cohort conducted in 2016, bone densitometry values were available for the baseline, 2nd, and 3rd follow-up surveys. From the 4th follow-up survey onwards, the participants living in Ansan did not test for BMD. Therefore, in this study, only the baseline and follow-up surveys from 2001–2005 could be used for analysis. The study was exempted from IRB review (IRB No. 2020GR0522), due to the use of public cohort data that could not identify the subjects.

### 2.2. Study Groups

#### 2.2.1. Longitudinal Observational Study

We designed a longitudinal observational study with participants having normal BMD at baseline for the analysis of newly developed osteopenia and/or osteoporosis during follow-up. Among them, the female cohort participants who had baseline T-scores > −1 were enrolled, and those who lacked urine electrolyte data and took prescribed steroid or anti-osteoporotic agents were excluded. We assessed whether the outcomes occurred within 2 follow-up surveys (within 4 years) in participants with normal BMD.

#### 2.2.2. Cross-Sectional Study

A cross-sectional analysis was performed to simultaneously compare serum and urinary factors from participants with normal bone density, osteopenia, and osteoporosis. We compared baseline data from longitudinal study participants (with normal bone density) with data from participants who were diagnosed with osteopenia and osteoporosis at baseline. All participants for whom serum and urine laboratory data were available were included in the study.

### 2.3. Study Outcome

In the longitudinal study, the outcome was the development of osteopenia and osteoporosis on the 1st or 2nd follow-up survey. Osteopenia was defined by a T-score between −1 and −2.5, and osteoporosis was defined by a T-score < −2.5. 

According to the guidelines of the International Society for Clinical Densitometry (ISCD) [15], the diagnosis of osteoporosis in premenopausal women depends on the Z-score rather than T-score. The International Osteoporosis Foundation (IOF), however, recommends the use of T-scores in those aged 20–50 years rather than the Z-score [16]. We assessed the Z-scores for premenopausal participants; however, none had a Z-score below −2.0 at follow-up surveys. Therefore, based on the definition of osteoporosis by the IOF, we defined premenopausal osteoporosis by the same T-score as postmenopausal osteoporosis.

### 2.4. Data Collection

We collected demographic and social characteristics, such as age, body mass indices, waist–hip ratio, alcohol intake history, smoking history, and the medical histories of participants, including hypertension, diabetes mellitus, and myocardial infarction. Baseline serum and urine laboratory tests were also performed. The estimated glomerular filtration rate (eGFR) was calculated using the CKD–EPI formula. Serum calcium was corrected by the serum albumin level using the formula: measured serum calcium + 0.8 × (4.0-serum albumin). For the adjustment of data from randomly sampled urine, urine sodium, calcium, uric acid, and protein levels were divided by urine creatinine level. 

Participants’ menopausal status was collected from the answers regarding self-assessed menopausal status at baseline and 1st and 2nd follow-up surveys. In the survey, female participants answered questions about their menopausal status and the age of menopause transition if they were going through menopause. 

Bone densitometry results from the baseline and follow-up surveys were also collected. A quantitative ultrasonography was performed to measure bone density (Omnisense 7000s, Sunlight Medical Ltd., Ramat Gan, Israel). The measurement sites were the distal radius and the mid-tibia of the non-dominant arm and leg, respectively. The average value of three repetitive measurements was taken as the final value [17,18]. The diagnosis of osteopenia and/or osteoporosis was based on the lower of the T-scores from the two measured sites.

### 2.5. Statistical Analyses 

The baseline continuous variables are shown as means with standard deviations or medians with interquartile ranges according to the normality test. The baseline categorical variables are presented as numbers and percentages. Comparison between groups was performed using a *t*-test or Wilcoxon signed rank test for continuous variables, and a Chi-square test or Fisher’s exact test for categorical variables. Univariate and multivariable multinomial logistic analyses were conducted for a risk analysis of the outcomes. The adjusted co-variables in multivariable analysis were chosen with the variable showing a *p*-value < 0.1 in univariate analysis. The cubic spline analysis was assessed using the mkspline function in Stata [19]. A spline curve is a general tool for interpolating points for plotting, and the third-degree polynomial—known as a cubic polynomial—is the one that is most typically chosen for constructing smooth curves [20,21]. All spline analyses were adjusted for age and baseline bone densitometry T-scores, according to the univariate logistic analysis results. For the cross-sectional analysis, variables across the three groups of participants according to bone densitometry were compared using the Kruskal–Wallis test. Statistical significance was set at *p* < 0.05. All analyses were performed using Stata version 15.0. 

## 3. Results

### 3.1. Baseline Characteristics of Longitudinal Cohort

**Figure 1:**
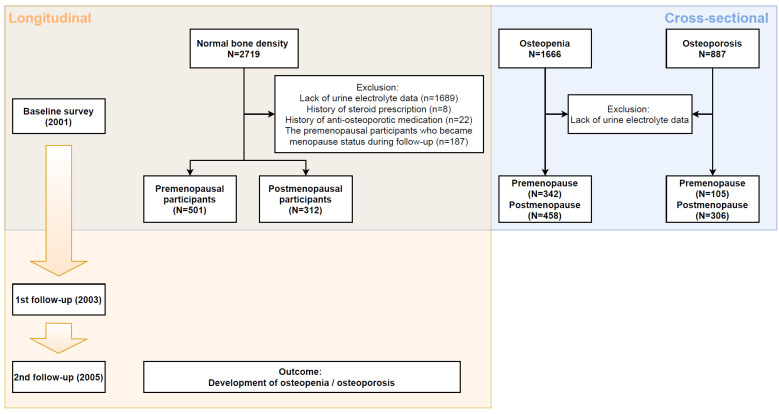
Study flow chart. Orange and blue squares represent longitudinal and cross-sectional study, respectively.

Among the 5272 female participants in the KoGES cohort, 2719, 1666, and 887 had normal BMD, osteopenia, and osteoporosis, respectively. After exclusions for lack of data, use of steroids, or anti-osteoporosis medication, 1000 patients with normal BMD at baseline were retained. According to menopause status, 688 patients had premenopausal status at baseline, and 187 of them had reached menopause at the 4-year follow-up. For the clarity of hormonal effects on bone, we only included and analyzed 501 participants who were in premenopausal status for 4 years and 312 participants who were already in postmenopausal status at baseline. A detailed flowchart of the study, including the criteria, is shown in Figure 1. 

The median ages of premenopausal and postmenopausal participants were 43 and 55 years, respectively. Most of the blood and urine electrolyte levels, along with medical histories, were significantly different across premenopausal and postmenopausal participants. Postmenopausal participants had higher serum and urine calcium levels and higher levels of uricosuria and proteinuria (Table 1). 

### 3.2. Risk Factors for Osteopenia and Osteoporosis in Pre- and Postmenopausal Participants in the Longitudinal Analysis

During the 4 years of follow-up, 64 participants developed osteoporosis and 354 developed osteopenia. In premenopausal participants, multivariable multinomial logistic regression analysis revealed age, baseline BMD, and serum calcium to be associated with the risk of osteopenia and/or osteoporosis. On the contrary, age, baseline BMD, hypertension, urine uric acid/creatinine, and serum calcium were associated with the risk of osteopenia and/or osteoporosis in postmenopausal participants (Table 2). 

**Figure 2:**
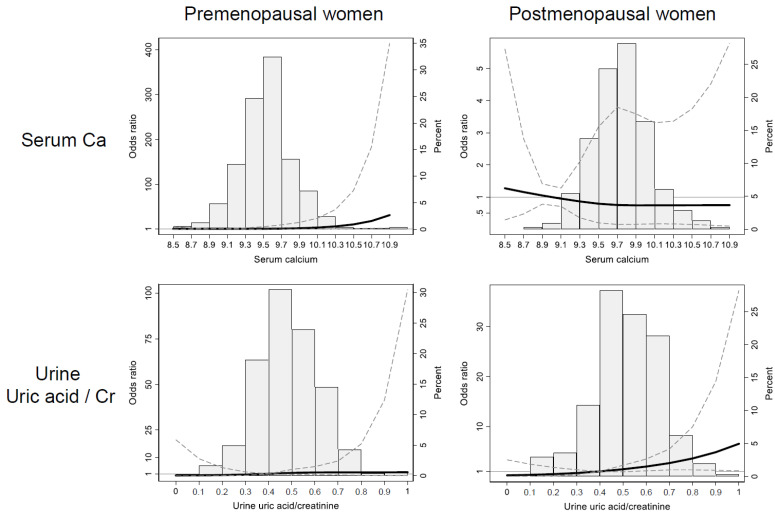
Multivariate-adjusted restricted cubic spline analysis of the effects of serum calcium (**upper row**) or urine uric acid levels (**lower row**) on osteoporosis risk in premenopausal (**left**) and postmenopausal (**right**) women. The bar plots show the frequency of patients on each value. The thick solid line shows the odds ratio, and the dashed line represents a 95% confidence interval. Abbreviations: Ca, calcium; Cr, creatinine).

To clarify the effect of urine uric acid and serum calcium on the risk of osteoporosis, cubic spline curves of odds ratios for osteoporosis were drawn according to the continuous levels of variables. In spline curves, the elevation of serum calcium over 10.5 increased the risk of osteoporosis only in premenopausal participants. In contrast, elevated uric acid over 0.6 in urine significantly increased the osteoporosis risk in postmenopausal participants, though not in premenopausal participants (Figure 2 and Table S1).

### 3.3. Difference of Serum Calcium, and Urine Uric Acid Levels between Bone Density Groups in Cross-Sectional Cohort

Serum calcium and urine uric acid levels, which were the identified risk factors in the longitudinal cohort study, were additionally evaluated among normal BMD, osteopenia, and osteoporosis participants in a cross-sectional cohort to determine whether the difference was maintained even after the development of bone loss.

In the normal BMD group, the same participants were used as in the longitudinal cohort, including 501 premenopausal and 312 postmenopausal participants. For the osteopenia and osteoporosis groups, among the excluded participants due to low BMD in the longitudinal study, 800 and 411 participants were enrolled, respectively, after exclusion of those who lacked urine electrolyte data (Figure 1). 

**Figure 3:**
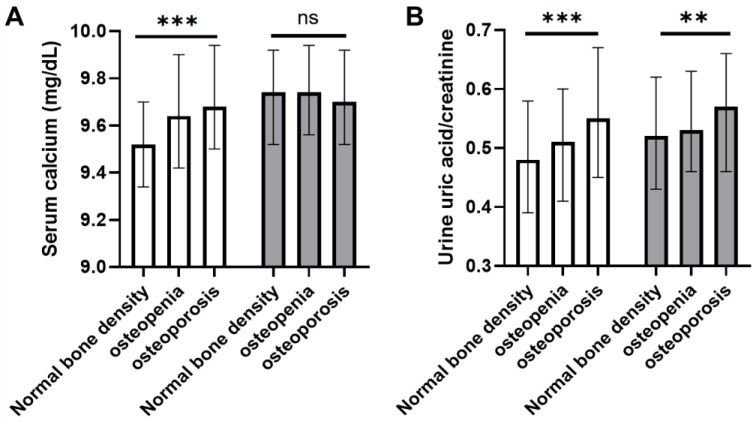
Serum calcium (**A**) and urine uric acid (**B**) levels according to bone densitometry values in the cross-sectional cohort. White bars and gray bars represent premenopausal and postmenopausal cohorts, respectively. ns, not significant; ***, *p* < 0.001. **, *p* < 0.01.

Analyzing the differences in serum calcium and urine uric acid levels in each group according to menopause status showed that serum calcium levels were significantly different, according to BMD, only in premenopausal but not in postmenopausal participants. However, urine uric acid levels showed a significant increase, with a decrease in BMD in both premenopausal and postmenopausal participants (*p* < 0.001 and *p* = 0.004, respectively; Figure 3). Differences in other clinical variables between groups were listed in Table S2.

## 4. Discussion

In this study, we identified biochemical markers that can predict bone loss, based on serum and urine electrolytes and biochemical values through longitudinal and cross-sectional analyses, in female participants. Corrected calcium level in serum and random uric acid level in urine, adjusted by creatinine, were found to be elevated even before the diagnosis of bone loss in premenopausal and postmenopausal women, respectively. Additionally, uric acid levels in urine were significantly elevated in both premenopausal and postmenopausal women with osteoporosis, suggesting a definite correlation between bone health and uric acid secretion in urine. 

Osteoporosis is a systemic bone disorder characterized by the loss of bone density and disorder of the bone microstructure. Several risk factors for osteoporosis have been revealed, such as age, sex, menopause, obesity, a family history of osteoporosis, and metabolic disorders related to bone health [22]. Although calcium metabolism is a critical factor in maintaining bone health, well-designed clinical research on the relationship between bone loss, calcium, and other electrolytes has been very limited [23,24,25]. 

Across the published studies on serum calcium levels and bone loss, results have been controversial. A longitudinal study with male participants showed men with high serum calcium levels had a higher risk of developing osteoporosis within 10 years [26]. In a cross-sectional study using NHANES data, serum calcium levels were negatively correlated with lumbar BMD [27]. On the other hand, a Mendelian randomization study reported that a genetic predisposition to increased serum calcium levels was not associated with increased BMD [23]. However, these studies did not consider the impact of gender differences and menopause status on bone metabolism. Female hormones are well known to play a significant role in the etiology of osteoporosis. Estrogen reduces bone turnover, suppresses bone resorption, and maintains bone formation [28,29]. Thus, sex differences and female hormonal status are the main factors that should always be considered in osteoporosis studies [6,7]. In the present study, we found serum calcium levels to increase the risk of bone loss, which is consistent with some previous studies [26,27], though only in premenopausal women. After the loss of estrogen effect, in postmenopausal women, the association between serum calcium and bone loss was reduced, and other risk factors of osteoporosis arose with hormonal changes.

In postmenopausal women, where the protective effect of hormones is weakened, uric acid in urine showed a higher association with the risk of osteoporosis development compared to the calcium levels in serum or urine. In addition, uric acid levels in urine were higher in both premenopausal and postmenopausal women who had already developed osteoporosis than in healthy controls with normal bone density. Recently, uric acid has emerged as an antioxidant in several studies [30]. However, uric acid is an antioxidant only in a hydrophilic environment, and it can be shifted to an oxidant in hydrophobic conditions [31,32]. Uric acid can increase nicotinamide adenine dinucleotide phosphate oxidase activity directly and enhance intracellular superoxide generation [32]. Furthermore, reactions of uric acid with oxidants may produce other radicals that can induce cellular injury [32,33]. Reduced bone density and osteoporosis have been found to be related to reduced levels of circulating antioxidants and increased oxidative stress, which inhibit osteoblastogenesis and bone formation [30]. Serum uric acid levels have been studied in various ways for bone health [30,33,34,35]; however, urine uric acid levels have not been studied to date. To the best of our knowledge, only one study has previously reported that there was no difference in uric acid levels in 24 h collected urine between individuals with osteoporosis and individuals with normal bone density [36]. However, since the study only compared cross-sectional data without adjusting for other clinical factors, there remains a question about the certainty and statistical significance of the results. Elevated excretion of uric acid in the present study may indicate a high total uric acid concentration in the body. Uric acids induce the production of inflammatory cytokines and cause oxidative stress, resulting in elevated osteoclast activity and decreased osteoblast viability [31,37]. Furthermore, uric acid inhibits 1α-hydroxylase expression in renal proximal tubules and thereby reduces 1,25(OH) vitamin D concentrations, which regulate osteoblast function and osteoclast differentiation [38]. Our study suggests that uric acid levels in urine that have been elevated for several years before osteoporosis need to be studied further as an important factor predicting the risk of bone loss, especially in postmenopausal women.

Although this study has great significance in providing important evidence about the possible association between rarely studied serum and urine biochemical factors and bone loss (even a few years before progression to osteoporosis), it has several limitations. First, some of the important biochemical measurements, such as parathyroid hormone and serum uric acid levels, were not available due to lack of data. Parathyroid hormone levels are related to the clearance of uric acid [39], and high levels of uric acid are known to be associated with the deficiency of vitamin D, an important factor for bone health [40,41]. Furthermore, it was difficult to determine whether the increase in uric acid levels in the urine of patients with osteoporosis was caused by excessive uric acid excretion due to an overall increase in uric acid, or by an abnormal loss via urine despite a normal amount of circulating uric acid in serum. Furthermore, data for well-known bone remodeling markers, such as procollagen-1 N-terminal peptide and C-terminal telopeptides of type I collagen were not available. Second, unrevealed confounding factors affecting bone loss may remain. Though we excluded the participants who had a history of steroid usage, the unknown confounding effect of steroid hormones, which are a known important factor in the development of osteoporosis [42], may have remained because of the lack of data for steroid hormone levels. In addition, the effect of other medications or health supplements, such as multivitamins including vitamin D, may have been overlooked. 

Osteoporosis, the clinical consequence of dysregulation of bone metabolism, causes various comorbidities, such as fractures, frailty, and deterioration of quality of life in elderly patients, and studies to elucidate the pathophysiology and etiology of osteoporosis are still in progress. In this study, we sought to discover early biomarkers for osteoporosis and osteopenia, focusing on the biochemical measurements of various urine and serum electrolytes that were previously undervalued. Our study suggests that dysregulation of uric acid metabolism may be one of the multifactorial processes in the development of osteoporosis. Uric acid levels in urine, as an easily measurable biomarker of bone loss, should be further evaluated in prospective studies.

## Figures and Tables

**Table 1:** Baseline characteristics of premenopausal and postmenopausal participants.

Variables	Premenopausal Women	Postmenopausal Women	
	*n* = 501	*n* = 312	*p*-Value
Age, years	43 (41–45)	55 (52–61)	<0.001
Body mass index, kg/m^2^	23.9 (22.2–26.0)	24.4 (22.6–26.7)	0.034
Waist-hip ratio, cm/cm	0.83 (0.78–0.89)	0.89 (0.83–0.94)	<0.001
Hypertension, *n* (%)	30 (6.0%)	59 (18.9%)	<0.001
Diabetes mellitus, *n* (%)	17 (3.4%)	23 (7.4%)	0.011
Myocardial infarction, *n* (%)	3 (0.6%)	5 (1.6%)	0.159
Alcohol habit, *n* (%)			0.016
Never drinker	331 (66.1%)	237 (76.0%)	
Ex-drinker	12 (2.4%)	7 (2.2%)	
Current drinker	148 (29.5%)	65 (20.8%)	
Smoking habit, *n* (%)			0.346
Never smoker	468 (93.4%)	296 (94.9%)	
Ex-smoker	3 (0.6%)	1 (0.3%)	
Current smoker	12 (2.4%)	13 (4.2%)	
Serum albumin, g/dL	4.1 (3.9–4.1)	4.1(3.9–4.1)	0.506
Serum blood urea nitrogen, mg/dL	12.3 (10.3–14.6)	13.7 (11.6–16.1)	<0.001
Estimated GFR, mL/min/1.73m^2^	104.2 (98.5–105.7)	93.8 (83.3–97.9)	<0.001
Bone density at baseline			
distal radius T-score	1.2 (0.4–2.1)	0.5 (−0.1 to 1.25)	<0.001
distal radius Z-score	1.3 (0.5–2.1)	1.7 (1.0–2.6)	<0.001
midshaft tibia T-score	0.1 (−0.3–1.1)	0 (−0.6–0.6)	<0.001
midshaft tibia Z-score	0.3 (−0.2–1.2)	1.0 (0.4–1.7)	<0.001
Serum and Urine electrolytes			
Serum calcium (albumin corrected), mg/dL	9.5 (9.3–9.7)	9.7 (9.5–9.9)	<0.001
Serum sodium, mmol/L	142 (140–143)	143 (141–144)	<0.001
Urine calcium/creatinine, mg/mg	0.12 (0.08–0.17)	0.14 (0.09–0.20)	<0.001
Urine sodium/creatinine, mmol/mg	1.51 (1.08–2.16)	1.93 (1.30–2.60)	<0.001
Urine uric acid/creatinine, mg/mg	0.48 (0.40–0.58)	0.52 (0.43–0.62)	<0.001
Urine protein/creatinine, mg/mg	0.06 (0.04–0.11)	0.08 (0.05–0.14)	<0.001

Abbreviations: GFR, glomerular filtration rate.

**Table 2:** The relative risk of developing osteopenia or osteoporosis compared to maintaining normal bone density according to each risk factor analyzed by multinomial multivariable logistic regression.

Variables	Osteopenia (Compared to Normal Bone Density)		Osteoporosis (Compared to Normal Bone Density)	
	RRR (95% CI)	*p*-Value	RRR (95% CI)	*p*-Value
In premenopausal women *				
Age, years	1.13 (1.06–1.21)	<0.001	1.15 (1.04–1.28)	0.001
Baseline T-score	0.38 (0.27–0.53)	<0.001	0.12 (0.03–0.44)	0.009
Serum calcium, mg/dL	0.90 (0.46–1.75)	0.752	4.03 (1.09–14.93)	0.037
In postmenopausal women **				
Age, years	1.03 (0.99–1.08)	0.169	1.07 (1.00–1.14)	0.043
Baseline T-score	0.33 (0.21–0.52)	<0.001	0.19 (0.09–0.40)	<0.001
Hypertension	2.30 (1.02–5.19)	0.044	3.83 (1.43–10.25)	0.008
Urine uric acid/creatinine, mg/mg	2.47 (0.38–16.20)	0.345	24.08 (1.79–323.69)	0.016
Serum calcium, mg/dL	3.33 (1.34–8.32)	0.01	1.77 (0.48–6.59)	0.395

Only variables showed *p*-value under < 0.05 in multivariable analysis were listed. Abbreviations: RRR, relative risk ratio; CI, confidence interval. * Adjusted by the variables showed *p*-value under 0.1 in univariable analysis; blood urea nitrogen, body mass index, estimated GFR and waist–hip ratio. ** Adjusted by the variables showed *p*-value under 0.1 in univariable analysis; diabetes mellitus, blood urea nitrogen and waist–hip ratio.

## Data Availability

The data supporting the findings of this study are available in clinical database from Korean Genome and Epidemiology Study and are accessible after permission from http://is.cdc.go.kr (accessed on 11 January 2021).

## Supplementary Materials

The following are available online at https://www.mdpi.com/article/10.3390/jcm10102155/s1, Table S1: The risk of osteoporosis according to serum calcium and urine uric acid in cubic spline analysis; Table S2. The clinical variables according to bone density groups in the cross-sectional cohort stratified by the menopausal status.
