# Supplementary material for: Urine and Serum Electrolytes and Biochemical Values Associated with Osteoporosis in Premenopausal and Postmenopausal Women: A Longitudinal and Cross-Sectional Study Using Korean Genome and Epidemiology Study (KoGES) Cohort
Source: J Clin Med. 2021 May 17;10(10):2155. doi: 10.3390/jcm10102155 (PMC8156403; doi:10.3390/jcm10102155)
Supplement: Supplementary file 1 [file jcm-10-02155-s001.zip › jcm-1152200-supplementary.pdf]

Table S1. The risk of osteoporosis according to serum calcium and urine uric acid in cubic spline analysis

Variables	premenopausal participants			postmenopausal participants		
	OR	LCI	UCI	OR	LCI	UCI
serum calcium						
8.5	0.99	0.17	5.74	1.27	0.29	5.61
8.7	0.99	0.34	2.85	1.16	0.48	2.81
8.9	1	0.7	1.42	1.05	0.78	1.41
9	1 (ref)	1	1	1 (ref)	1	1
9.1	1	0.71	1.43	0.95	0.71	1.28
9.3	1.01	0.35	2.9	0.86	0.36	2.1
9.5	1.08	0.21	5.65	0.79	0.2	3.18
9.7	1.38	0.2	9.72	0.75	0.15	3.8
9.9	2.17	0.31	15.17	0.74	0.15	3.59
10.1	3.69	0.55	24.58	0.74	0.17	3.32
10.3	6.3	0.9	43.98	0.75	0.17	3.36
10.5	10.76	1.34	86.51	0.75	0.15	3.75
10.7	18.39	1.84	183.86	0.75	0.12	4.52
10.9	31.43	2.39	414.08	0.75	0.1	5.78
Urine uric acid/creatinine						
0	0.11	0	19.68	0.21	0.01	3.33
0.1	0.19	0	9.27	0.31	0.04	2.45
0.2	0.33	0.02	4.37	0.46	0.12	1.81
0.3	0.58	0.16	2.06	0.68	0.35	1.33
0.4	1 (ref)	1	1	1 (ref)	1	1
0.5	1.5	0.67	3.39	1.44	0.92	2.24
0.6	1.75	0.62	4.94	1.98	1.12	3.49
0.7	1.78	0.4	8.03	2.67	1.3	5.5
0.8	1.8	0.19	17.42	3.6	1.31	9.94
0.9	1.82	0.08	41.43	4.86	1.24	19.02
1	1.84	0.03	101.94	6.54	1.15	37.25

Abbreviations: OR, odds ratio; LCI, lower bound of 95% confidence interval; UCI, upper bound of 95% confidence interval.

Table S2. The clinical variables according to bone density groups in cross-sectional cohort stratified by the menopausal status.

(A) Premenopausal women

Variables	Normal bone density<br>n = 501	Osteopenia<br>n = 342	Osteoporosis<br>n =105	*p*-Value
Age, years	43 [41–45]	49 [44-60]	61 [55-65]	<0.001
Body mass index, kg/m^2	23.9 [22.2–26.0]	25.0 [23.0-27.0]	25.7 [23.7-27.3]	<0.001
Waist-hip ratio, cm/cm	0.83 [0.78–0.89]	0.86 [0.80-0.93]	0.89 [0.85-0.95]	<0.001
Hypertension, *n* (%)	30 (6.0%)	53 (15.5%)	26 (24.8%)	<0.001
Diabetes mellitus, *n* (%)	17 (3.4%)	26 (7.6%)	2 (1.9%)	0.007
Myocardial infarction, *n* (%)	3 (0.6%)	2 (0.6%)	1 (1.0%)	0.909
Alcohol habit, *n* (%)				0.001
Never drinker	331 (66.1%)	246 (71.9%)	77 (73.3%)	
Ex-drinker	12 (2.4%)	10 (2.9%)	8 (7.6%)	
Current drinker	148 (29.5%)	77 (22.5%)	16 (15.2%)	
Smoking habit, *n* (%)				0.626
Never smoker	468 (93.4%)	311 (90.9%)	94 (89.5%)	
Ex-smoker	3 (0.6%)	2 (0.6%)	1 (1.0%)	
Current smoker	12 (2.4%)	13 (3.8%)	5 (4.8%)	
Serum albumin, g/dL	4.1 [3.9–4.1]	4.0 [3.9-4.1]	4.0 [3.9-4.1]	0.14
Serum blood urea nitrogen, mg/dL	12.3 [10.3–14.6]	12.9 [11.0-15.5]	13.6 [11.7-16.6]	<0.001
Estimated GFR, mL/min/1.73m^2	104.2 [98.5–105.7]	97.9 [88.1-103.5]	91.9 [88.7-96.5]	<0.001
Serum and Urine electrolytes				
Serum calcium (albumin corrected), mg/dL	9.5 [9.3–9.7]	9.6 [9.4-9.9]	9.7 [9.5-10.0]	<0.001
Serum sodium, mmol/L	142 [140–143]	142 [141-144]	143 [141-144]	<0.001
Urine calcium/creatinine, mg/mg	0.12 [0.08–0.17]	0.13[0.08-0.19]	0.16 [0.11-0.24]	<0.001

Urine sodium/creatinine, mmol/mg	1.51 [1.08–2.16]	1.60 [1.11-2.33]	1.95 [1.4-2.5]	<0.001
Urine uric acid/creatinine, mg/mg	0.48 [0.40–0.58]	0.51 [0.41-0.60]	0.56 [0.47-0.68]	<0.001
Urine protein/creatinine, mg/mg	0.06 [0.04–0.11]	0.07 [0.04-0.13]	0.08 [0.05-0.14]	<0.001

(B) Postmenopausal women

**Variables**	**Normal bone density**<br>**n = 312**	**Osteopenia**<br>**n = 458**	**Osteoporosis**<br>**n =306**	***p*-Value**
Age, years	55 [52–61]	61 [56-65]	61 [56-66]	<0.001
Body mass index, kg/m²	24.4 [22.6–26.7]	25.1 [23.1-27.2]	25.3 [22.9-28.0]	0.046
Waist-hip ratio, cm/cm	0.89 [0.83–0.94]	0.92 [0.87-0.97]	0.92 [0.87-0.98]	<0.001
Hypertension, *n* (%)	59 (18.9%)	119 (26.0%)	75 (24.5%)	0.065
Diabetes mellitus, *n* (%)	23 (7.4%)	50 (10.9%)	17 (5.6%)	0.023
Myocardial infarction, *n* (%)	5 (1.6%)	3 (0.7%)	3 (1.0%)	0.439
Alcohol habit, *n* (%)				0.559
Never drinker	237 (76.0%)	349 (76.2%)	238 (77.8%)	
Ex-drinker	7 (2.2%)	16 (3.5%)	6 (2.0%)	
Current drinker	65 (20.8%)	81 (17.7%)	56 (18.3%)	
Smoking habit, *n* (%)				0.121
Never smoker	296 (94.9%)	411 (89.7%)	278 (90.8%)	
Ex-smoker	1 (0.3%)	9 (2.0%)	1 (0.3%)	
Current smoker	13 (4.2%)	20 (4.4%)	14 (4.6%)	
Serum albumin, g/dL	4.1 [3.9–4.1]	4.1 [3.9–4.1]	4.1 [3.9–4.1]	0.764
Serum blood urea nitrogen, mg/dL	13.7 [11.6–16.1]	14.1 [12.0-16.3]	13.9 [11.7-16.8]	0.339
Estimated GFR, mL/min/1.73m²	93.8 [83.3–97.9]	90.6 [81.0-94.5]	91.2 [87.5-95.1]	<0.001
Serum and Urine electrolytes				

Serum calcium (albumin corrected), mg/dL	9.7 [9.5–9.9]	9.7 [9.6-9.9]	9.7 [9.5-9.9]	0.449
Serum sodium, mmol/L	143 [141–144]	143 [142–144]	143 [141–144]	0.347
Urine calcium/creatinine, mg/mg	0.14 [0.09–0.20]	0.14 [0.90-0.22]	0.14 [0.09-0.21]	0.656
Urine sodium/creatinine, mmol/mg	1.93 [1.30–2.60]	1.86 [1.32-2.60]	1.92 [1.27-2.73]	0.986
Urine uric acid/creatinine, mg/mg	0.52 [0.43–0.62]	0.53 [0.46-0.63]	0.57 [0.45-0.65]	0.014
Urine protein/creatinine, mg/mg	0.08 [0.05–0.14]	0.08 [0.05-0.13]	0.09 [0.05-0.15]	0.426

Abbreviations: GFR, glomerular filtration rate.
